# Post-seismic deformation mechanism of the July 2015 MW 6.5 Pishan earthquake revealed by Sentinel-1A InSAR observation

**DOI:** 10.1038/s41598-020-75278-0

**Published:** 2020-10-28

**Authors:** Sijia Wang, Yongzhi Zhang, Yipeng Wang, Jiashuang Jiao, Zongtong Ji, Ming Han

**Affiliations:** 1 College of Geology Engineering and Geomatics, Chang’an University, Xi’an, 710054 China; 2 State Key Laboratory of Geo-Information Engineering, Xi’an, 710054 China

**Keywords:** Natural hazards, Geodynamics

## Abstract

On 3 July 2015, the Mw 6.5 Pishan earthquake occurred at the junction of the southwestern margin of the Tarim Basin and the northwestern margin of the Tibetan Plateau. To understand the seismogenic mechanism and the post-seismic deformation behavior, we investigated the characteristics of the post-seismic deformation fields in the seismic area, using 9 Sentinel-1A TOPS synthetic aperture radar (SAR) images acquired from 18 July 2015 to 22 September 2016 with the Small Baseline Subset Interferometric SAR (SBAS-InSAR) technique. Postseismic LOS deformation displayed logarithmic behavior, and the temporal evolution of the post-seismic deformation is consistent with the aftershock sequence. The main driving mechanism of near-field post-seismic displacement was most likely to be afterslip on the fault and the entire creep process consists of three creeping stages. Afterward, we used the steepest descent method to invert the afterslip evolution process and analyzed the relationship between post-seismic afterslip and co-seismic slip. The results witness that 447 days after the mainshock (22 September 2016), the afterslip was concentrated within one principal slip center. It was located 5–25 km along the fault strike, 0–10 km along with the fault dip, with a cumulative peak slip of 0.18 m. The 447 days afterslip seismic moment was approximately 2.65 × 10^17^ N m, accounting for approximately 4.1% of the co-seismic geodetic moment. The deep afterslip revealed that a creeping process from steady-state “secondary” creeping to accelerating “tertiary” creep in the deep of fault. The future seismic hazard deserves further attention and research.

## Introduction

**Figure 1:**
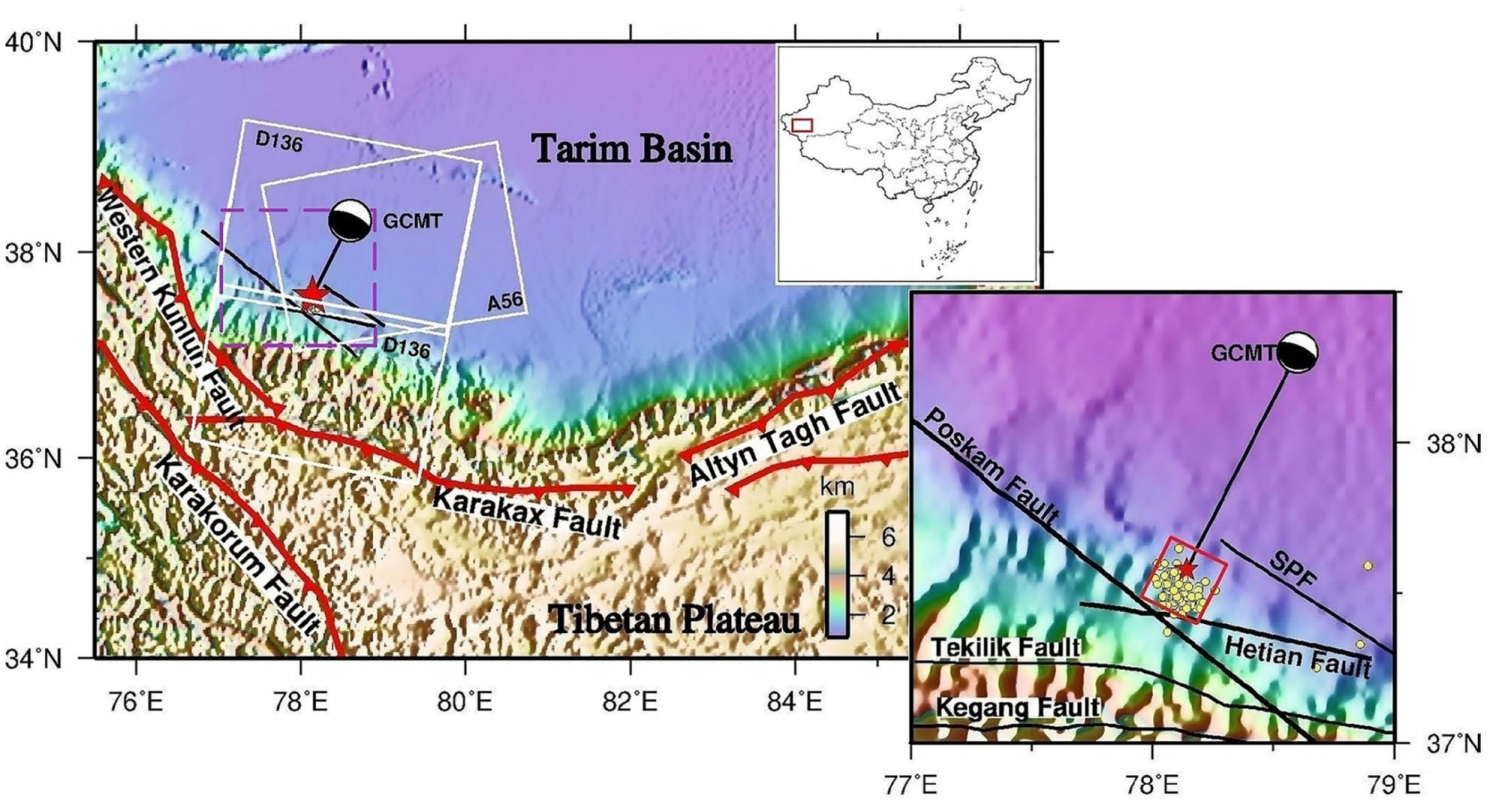
The research regional tectonic setting. The purple dashed rectangle in accord to the zoomed- in the area in the smaller map. The GCMT catalog focal mechanism of the 2015 Mw 6.5 Pishan earthquake. The purple dashed rectangle corresponds to the zoomed-in area in the smaller map. The position of the mainshock is denoted by the red star. The aftershocks with M ≥ 3.0 up to 447 days after the mainshock are represented by yellow circles^16^. The black lines are faults (SPF: Southeast Pishan Fault). White rectangles represent the territory covered of the Sentinel-1A SAR images from ascending Path56 (A56) and descending Path136 (D136). The rectangular flat of fault projected on the ground is denoted by the red rectangle. Entire shocks in the figure are available on the China Earthquake Data Centre (https://data.earthquake.cn/data/).

At 09:07 UTC + 8 on 3 July 2015, an M_W_ 6.5 earthquake occurred at Pishan in Xinjiang (Fig. 1). The epicenter was located in the West Kunlun fault belt, at the junction of the southwestern margin of the Tarim Basin and the northwestern margin of the Tibetan Plateau^1,2^. Since the Kunlun Mountains Mw 8.1 earthquake occurred in 2001, a series of extremely destructive earthquakes have occurred around the Tibetan Plateau, such as the Mw 7.3 earthquake in the Yutian area in 2008 and 2014^3^, respectively. Both the earthquakes and the Pishan earthquake hit in the junction zone between the west Kunlun fault and the Altyn Tagh fault. As shown in Fig. 1, the northern margin of the Tibetan Plateau in western China reveals the profile of the Tarim Basin^1^. The western Kunlun Fault reaches the northwest, the Altyn Tagh Fault reach the southeast encompass the Tarim Basin^4^. The strike-slip Karakax fault was characterized by verging on the southern flank of the Tarim Basin^4,5^. This large strike-slip fault was considered by some scholars to be the western continuation of the Altyn Tagh fault, to sever through the foundation of the crust^4–6^. The Poskim fault was considered to be a hidden fault in the mountain front^3^. The Hetian fault belt in the southwest of the Tarim Basin, extending more than 300 km, was taken for one of the primary fault belts^6^. The Western Kunlun fault is characterized by tectonic deformation associated with reverse faults and folds, which coordinates with the relative movement between the Pamir front thrust belt and the West Kunlun-Tarim Block^7,8^. Zubovich et al.^9^ demonstrated that the Western Kunlun fault tectonic belt was dominated by compression, shortening, and deformation, and the dextral strike-slip rate was only 1–3 mm/a, forming a typical thin-skinned nappe tectonic system.

Based on the solved focal mechanism that a thrust rupture triggered the M_W_ 6.5 Pishan mainshock, for the Global Centroid Moment Tensor (CMT) Catalog, aftershocks of M_W_ 4.5 and M_W_ 4.6 successively struck 2 h after the mainshock. Zhang et al.^10^ used the digital broadband seismic data recorded by Xinjiang network stations to obtain the aftershock sequence, which extended unilaterally along an NWW direction with a spreading length of approximately 50 km. The mainshock fault inclines towards the SW, showing the characteristics of a shovel thrust fault^10^. Ainscoe et al.^11–13^ used InSAR data and seismic waveforms to solve the fault parameters, to inverse the slip distribution of the co-seismic deformation. It was revealed that the earthquake was induced by a preexisting slope, reverse‐dipping toward the Tibetan Plateau, the range of ∼9–13 km in deep blind fault^12,13^. The Pishan earthquake triggered the root faults to produce small swarms, but the previously accumulated tectonic stress was not completely released^14,15^. The future seismic hazard deserves further attention.

Since the co-seismic displacements recorded by InSAR include the influence of *M*_W_ 4.6 and *M*_W_ 4.5 aftershocks, the study of the co-seismic deformation and fracture mechanism of Pishan *M*_W_ 6.5 was insufficient to reveal the intrinsic dynamic correlation of mainshock and aftershocks.

Therefore, to further probe into the seismogenic condition and post-seismic deformation mechanism, we adopted Sentinel-1A data and employed the Small Baseline Subset InSAR (SBAS-InSAR) technique with the correction of atmospheric error and orbit error to extract the mainshock deformation within 447 days. Then the SDM method was applied to invert the afterslip distribution. The post-seismic deformation transmits pivotal feedback in the temporal evolution of the afterslip. A comparison of time evolution between the afterslip and the aftershocks was conducted to understand the mechanism of aftershock triggering. We comprehensively studied the relationship between afterslip and co-seismic slip to examine regional tectonic movement.

## Results

### Post-seismic deformation time series

**Figure 2:**
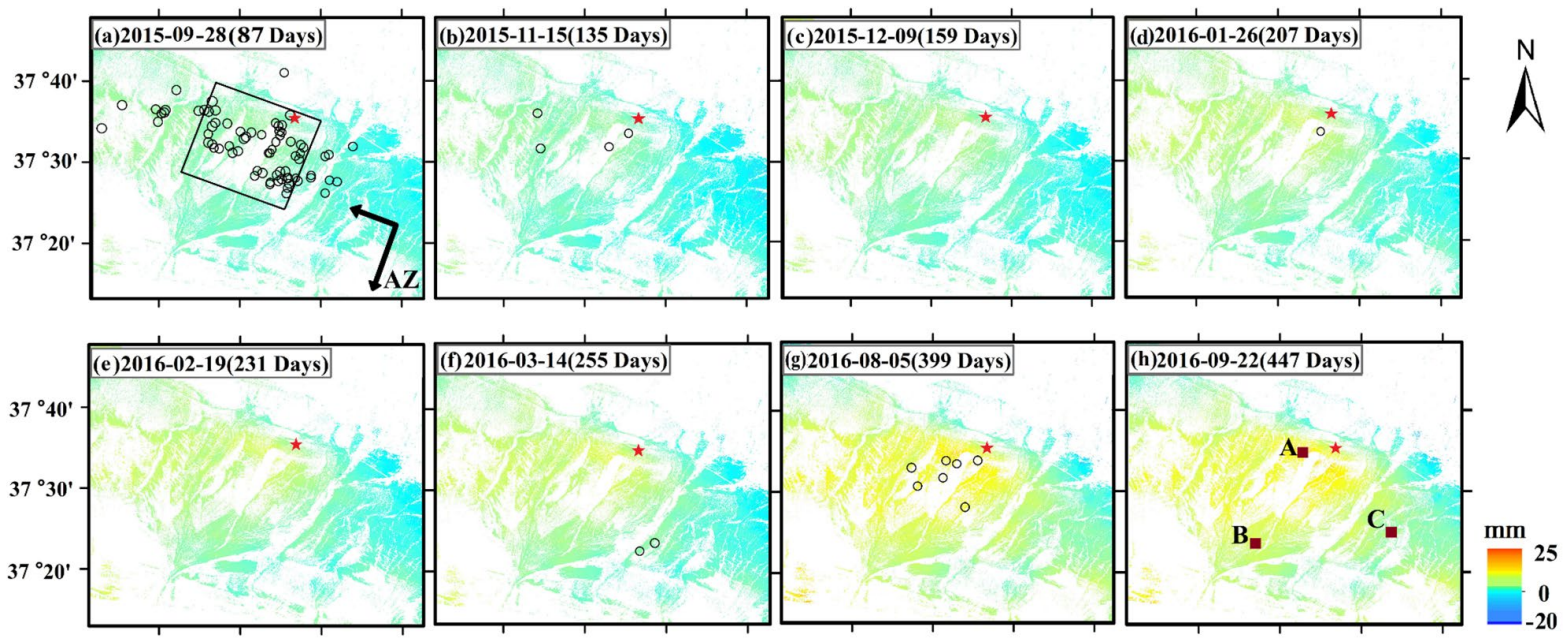
Time-series of deformation after the Mw6.5 Pishan earthquake. Positive values and negative values separately denote motion towards and far from the satellite. Days refer to the time counted from July 3, 2015. The epicenter is denoted by the red star. M_W_ $\ge$ 3.0 aftershocks in each time interval are represented by black circles. The rectangular flat of fault projected on the ground is denoted by the black rectangle.

The entire 9 SAR images were gathered from descending Path136 (hereafter referred to as D136). The earliest image acquisition time is 15 days after the earthquake, i.e., 18 July 2015, and the latest image is 22 September 2016. We calculated a time series and referenced it in time to the image acquired 15 days after the mainshock. Figure 2 shows the post-seismic deformation time series. Positive values indicate that the deformation approached the satellite in the direction of line-of-sight (LOS); negative values denote deformation in LOS motion turned aside away from the satellite. The aftershock dataset (M_W_ $\ge$ 3.0) after the mainshock within 480 days was assembled. Figure 2 plots the spatial distribution of the aftershock sequence. The aftershock distribution is most dense in the NWW direction (see Fig S1). The bottom of rupture fault portion remained locked that induced the Pishan earthquake^15^. A strand of aftershocks following the mainshock primarily focused on the Pishan anticline, which mirrored seismic events evoked by enhancing stress forbye the locked fault portion^10,13,15^.

We selected 9 dates of accumulated LOS deformation within 447 days of the mainshock, as shown in Fig. 2. The accumulated post-seismic deformation improved over time. At 2015-11-15 (135 days), InSAR has shown preponderance in measuring the apparent displacement signal after the mainshock, and its LOS-direction maximum positive values around 18 mm and negative values around -14 mm. At the 2016-09-22 (447 days), post-seismic displacement features were most prominent, with significant positive values of up to 25 mm and negative values of up to − 20 mm in the LOS-direction. Positive range occurred around the epicenter and negative range occurred in the southern far away from the epicenter. By analyzing the post-earthquake deformation field, we found that the deformation mainly occurred in the fault plane.

**Figure 3:**
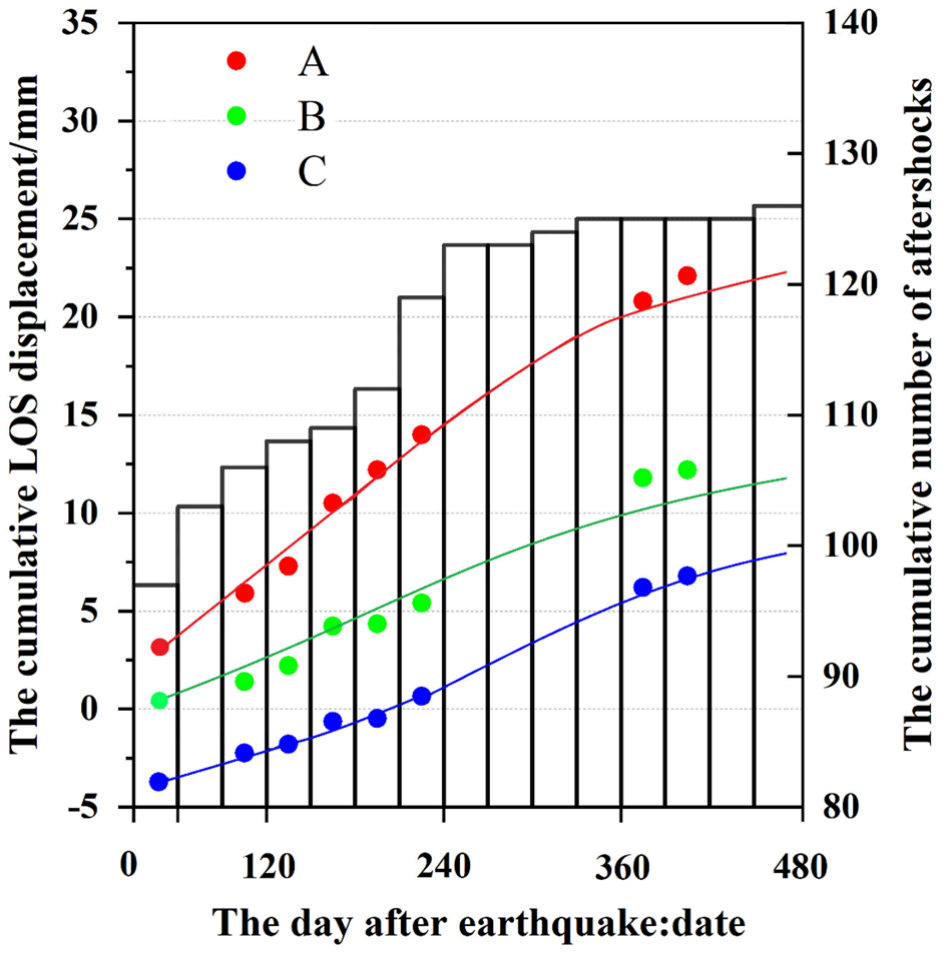
The LOS accumulated deformation and aftershocks over time. Patches A, B, C are separately indicated by red, green, and blue. The position (Patches A, B, and C) are displayed in Fig. 2(h). Points show the accumulated time-series deformation. The fit of the accumulated time-series deformation was shown by a solid line. The left Y-axis and the right Y-axis separately represent the deformation and the number of aftershocks. The X-axis shows the time after the earthquake.

Three patches (A, B, and C in Fig. 2h) were selected from the deformation time-series. To obtain a smoothed time series of deformation, we employed a logarithmic function to fit the discrete data of the three patches^17^:

1 $$ y = a{\log}_{10} \left( {1 + t} \right) $$

where $y$ denote the accumulated LOS deformation (mm), $a$ show the logarithmic decay for amplitude, and $t$ denote days of the post-earthquake time. Figure 3 depicts the curve fitting lines by a logarithmic function for patches A, B, and C.

A histogram in Fig. 3 shows aftershocks (≥ Mw 3.0) 480 days after the mainshock. As of August 3, 2015, 98 aftershocks(≥ Mw 3.0) occurred; subsequently, the rate of seismic events decreased sharply. Similarly, the time-series cumulative deformation shows the law of logarithmic function decay. The similarity between the InSAR observations and aftershock activity may both be driven by the same process. There are three main mechanisms for the deformation of the crust post-earthquake: afterslip, poroelastic rebound, and viscoelastic relaxation^17^. Viscoelastic relaxation is the deformation, happened at the middle and lower crust, attribute to the viscous flow of material. Afterslip is a creep slip dislocation on or around the earthquake generating fault caused by co-seismic rupture. In the short term, viscoelastic relaxation, generally speaking, does not play a dominant role in near-field displacement after the earthquake^17–19^. It is considered as poroelastic rebound when the direction of post-seismic motion is opposite to the motion of co-seismic, or displacement of post-earthquake decreased with the extension of time^20^. After the earthquake, we considered that the near-field displacement increased with the extension of time, which was contrary to the deformation characteristics caused by the poroelastic rebound. The above features are most likely caused by afterslip^20^. It is generally believed that deformation is adapted by cracked (fragile) processes over all the upper crust^21^. Whereas, ductile (plastic) deformation primarily throughout the lower crust, governed by thermally-induced dislocation creep^21^. The mainshock was a characteristic reverse thrust belt earthquake, not only induced folding of the upper crust but also leading to aftershocks on the Pishan anticlines^15^. The post-seismic deformation mainly occurred on the fault plane was a manifest of the stress change caused by co-seismic rupture. According to the phenomenon of afterslip and aftershocks, the temporal evolution of both are corresponding, Yang et al.^17^ believed that aftershocks were possessed by afterslip. Perfettini et al.^21^ found that post-seismic displacement and the speed of seismic events show a coetaneous decline in the region of aftershocks. For the upper crust, it was considered that the afterslip dominated the elastic deformation, giving rise to displacement change observed on the ground^21^. The temporal evolution of aftershocks and the displacement feature in the satellite LOS direction was corresponding with previous studies^17,19,21^. Similarly, we conceived that afterslip undertakes a major impact, in the short term, on near-field displacement after the Pishan earthquake.

### Characteristics of post-seismic afterslip distribution

**Figure 4:**
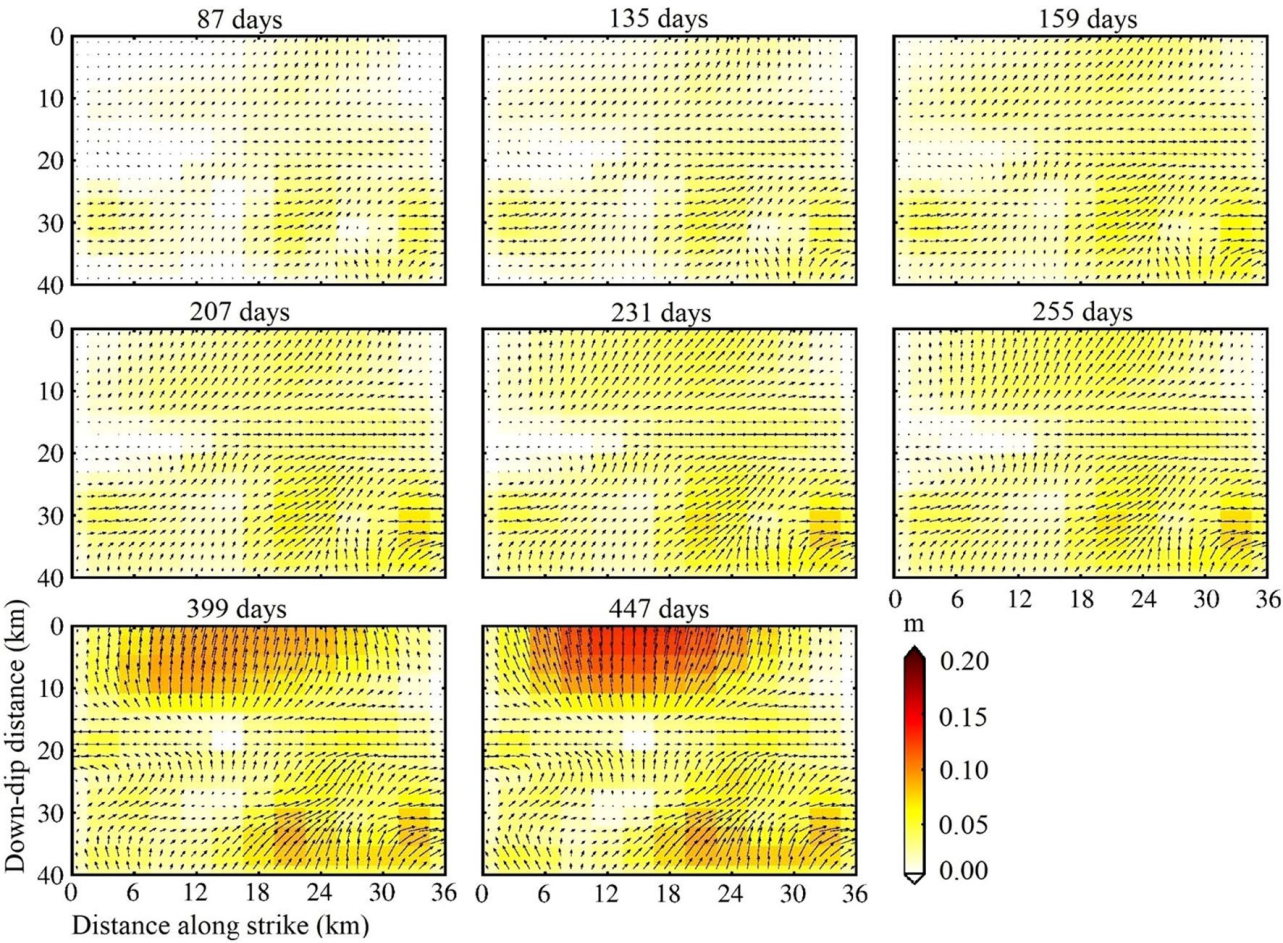
Calculating each date post-seismic SAR for accumulated transient afterslip. The horizontal axis and vertical axis are fault length along with the strike and fault depth along with the dip, respectively. The length and the direction of the arrows indicate the magnitude and the direction of afterslip, respectively.

From July 18, 2015, to September 22, 2016, We calculated the cumulative afterslip at 8 intervals, as shown in Fig. 4. We determined the slip direction by solving for slip along two vectors (strike vector and dip vector). The main feature of afterslip is thrusting, which is similar to the characteristics of the co-seismic slip^12,14^. According to the analysis of the afterslip (Fig. 4), the afterslip activity indicated that the shallow slip accumulated gradually up from days 87 to 447 of the mainshock in 0–10 km along with the fault dip, and the deep SSE slip in 30–40 km along with the fault dip accumulates more gradually over the entire period.

Theoretical and academic fault models were derived, some authors have penetrated research and performed triaxial “creep tests” to deduce the entire creep process^21–23^. There has been proved that the cycle of primary, secondary, and tertiary creep, repetitively, throughout the overall creep process^24^. The first period of “primary” creep is undergoing decelerating promptly, followed by stationary state “secondary” creep for a longer time, up to the accelerated process, “tertiary” creep eventually gives rise to a fault rupture by static fatigue; throughout the inter-seismic cycle, “secondary” semi-stable creep, in most cases, it is considered as the predominant process^21^. We should note that the afterslip was in a steady state before 255 days, which corresponded to some steady-state “secondary” creep. The aftershocks occurred continuously on the fault plane during this period. The static fatigue is that the crack or rupture grows slowly with time under the sustaining loading of the stress, which is a subcritical state of fault fracture^21–24^. When the fault slip comes through rate-and state friction laws on account of imbalance intrinsic, or static fatigue brittle creep spread and extend on the fault zone, it was considered the potential origin for crustal deformation-induced transiently^21^. The magnitude of afterslip in the shallow fault correspondingly increased from 399 to 447 days, which was consistent with some accelerating “tertiary” creep. The instability of this creeping process may lead to the aftershock on October 5, 2016. Our results also support the comment that aftershocks discontinuously or serially are induced prevailingly by afterslip^24,25^.

**Figure 5:**
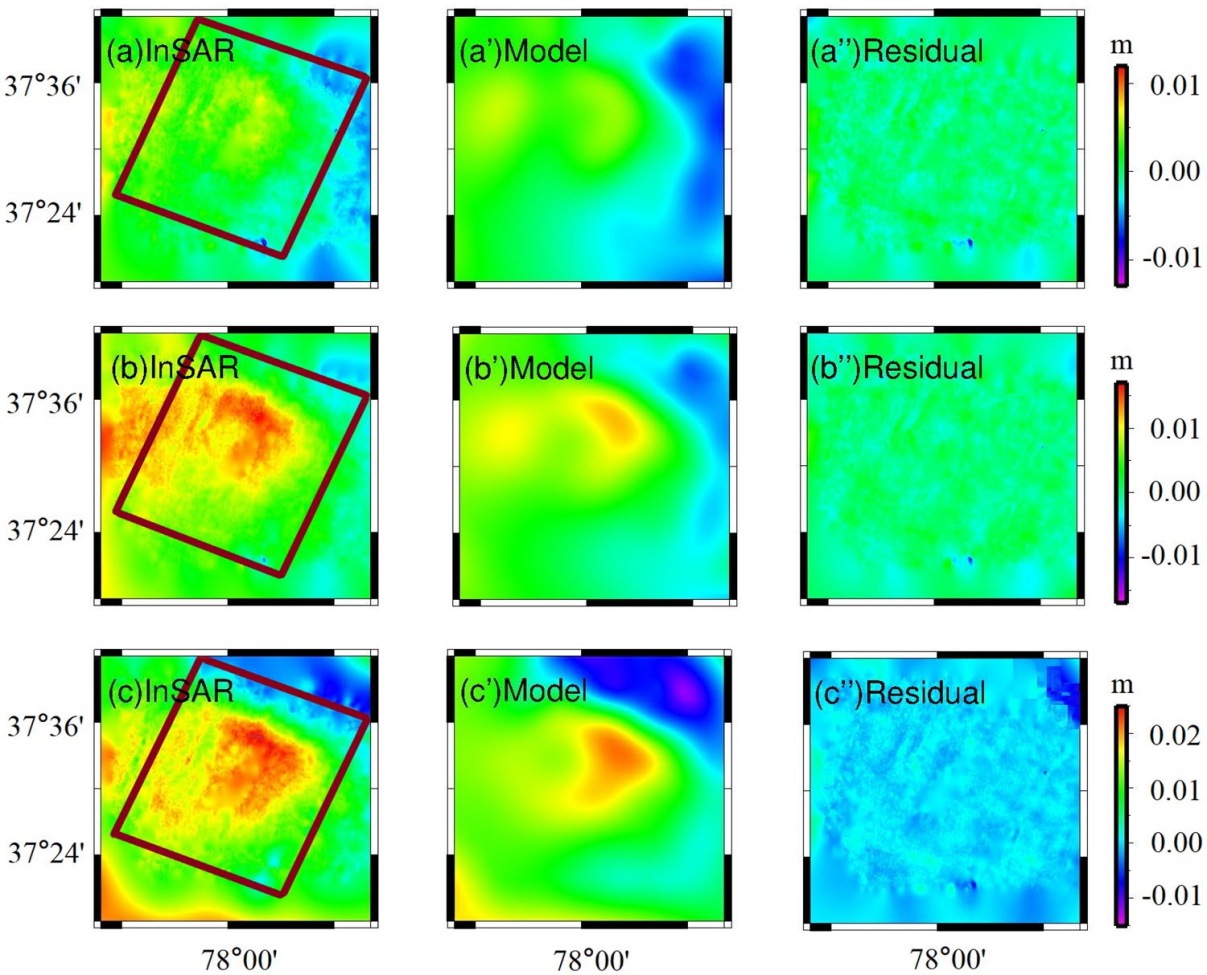
The simulated post-seismic deformation fields and the residuals are based on the afterslip. The brown rectangle represents the surface projection of the modeled fault. (**a**–**c**) are the InSAR deformation fields of 135, 255, 447 days after the earthquake, respectively; (**a**’–**c**’) is the corresponding simulated deformation fields, respectively; (**a**’’–**c**’’) is the corresponding residuals, respectively; and the other 5 dates of simulated deformation fields and residuals are similar.

At 447 days after the mainshock (September 22, 2016), the afterslip formed one slip center(Fig. 4). It was located 5–25 km along the fault strike, 0–10 km along with the fault dip, with a peak magnitude of 0.18 m. Along the fault strike at 18–35 km, along with the dip of 30–38 km, with a peak magnitude of 0.09 m. The simulated post-earthquake deformation fields and residuals are shown in Fig. 5. The simulated post-seismic deformation fields were similar to the post-seismic deformation fields measured by InSAR. The simulation residuals of the main deformation zone after the mainshock were usually small and the larger residuals were mainly distributed in the northeast corners (Fig. 5), which might be related to the viscoelastic relaxation effect after the mainshock or to tectonic motion changes.

Fig. S2 shows the time evolution of the cumulative post-seismic moment released by afterslip and CENC-recorded aftershocks. The curve fit the InSAR moment using an exponential equation:

2 $$ n\left( t \right) = k\left( {p - e^{ - ct} } \right) $$

where $k$, $p$, $c$ are constants, $n\left( t \right)$ is the cumulative post-seismic moment released by afterslip, and $t$ is the time after the earthquake (day).

When the shear modulus is 30 GPa, the afterslip moment of 87 days (28 September 2015) after the mainshock was approximately 0.5 $\times$ 10^17^ N m, the moment magnitude of statistical equivalently, for M_W_ 5.03 (Fig. S1); the afterslip moment of 447 days (September 22, 2016) after the mainshock was approximately 2.65 $\times$ 10^17^ N m, amounted to the moment magnitude M_W_ 5.5 (Fig. S2). The rate of the seismic moment released by aftershocks from CENC increased instantly until decayed overtime. The afterslip moment release evolves with exponential decay. It was indicated the afterslip moment is greater than the seismic moment here, to a mass of the post-seismic deformation is aseismic. Therefore, to explain the surface displacement estimated, for the upper crust, it is momentous due to deep afterslip dominates the elastic deformation^21^.

### Comprehensive analysis of post-seismic afterslip and co-seismic slip

Some scholars have made achievements in the study of the Pishan co-seismic slip distribution^12,14^ and iteratively searched the model parameters and inverted slip distribution of the constructed model. The same Sentinel-1A data as Wen et al.^14^ was used for coseismic deformation and coseismic slip inversion (Table S1). Two interferograms covering the region of the co-seismic deformation, from the ascending Path56 (for short A56) to descending Path136 (for short D136) were employed, Table S1 showed the data contents. To obtain co-seismic deformation interferograms, the two-pass InSAR approach, it was utilized for data processing^20^.

A method of acquiring upward and eastward units of surface deformation was imported, 2.5-dimensional (2.5-D)analysis technology, to process from ascending and descending LOS data ^\* MERGEFORMAT^^14^. $U$ as the LOS displacements to can be expressed as:

3 $$ U \approx U_{e} s_{e} + U_{u} s_{u} $$

where $U_{e}$ represent eastward displacement, $U_{u}$ represent upward displacement. Besides, in the east and vertical directions, separately, $s_{e}$ and $s_{u}$ show the direction cosines.

**Figure 6:**
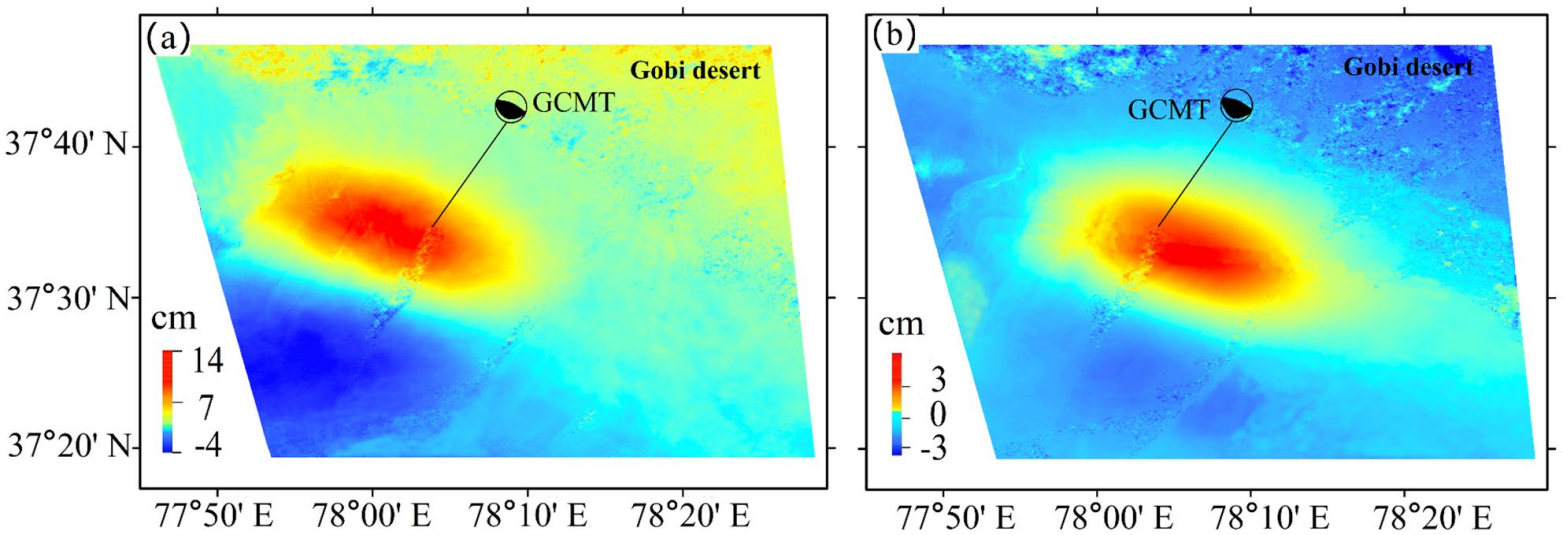
Calculated upward (**a**) and eastward (**b**) components for surface displacement from the Sentinel-1A data. The decorrelation noise was serious in the Gobi desert.

Figure 6 shows the surface deformation map (2.5-D) with upward (a) and eastward (b) components. In the eastward direction (Fig. 6b), the main red displacement zone toward moved eastward, with the top value eastward motion up to 6 cm; the blue displacement zone in the northwest corner with the supreme value closed to 3 cm toward westward. In the upward direction, the maximum uplift displacement (red region in Fig. 6a) was 14 cm, and the maximum subsidence (blue region in Fig. 6a) was 3.3 cm. In the Gobi desert, the random and decorrelation noise interference of InSAR measurement was relatively serious, and the co-seismic displacement error was large. We adopted the same crustal layering model Crust1.0 and fault model as post-seismic afterslip inversion (Tables S2 and S3).

**Figure 7:**
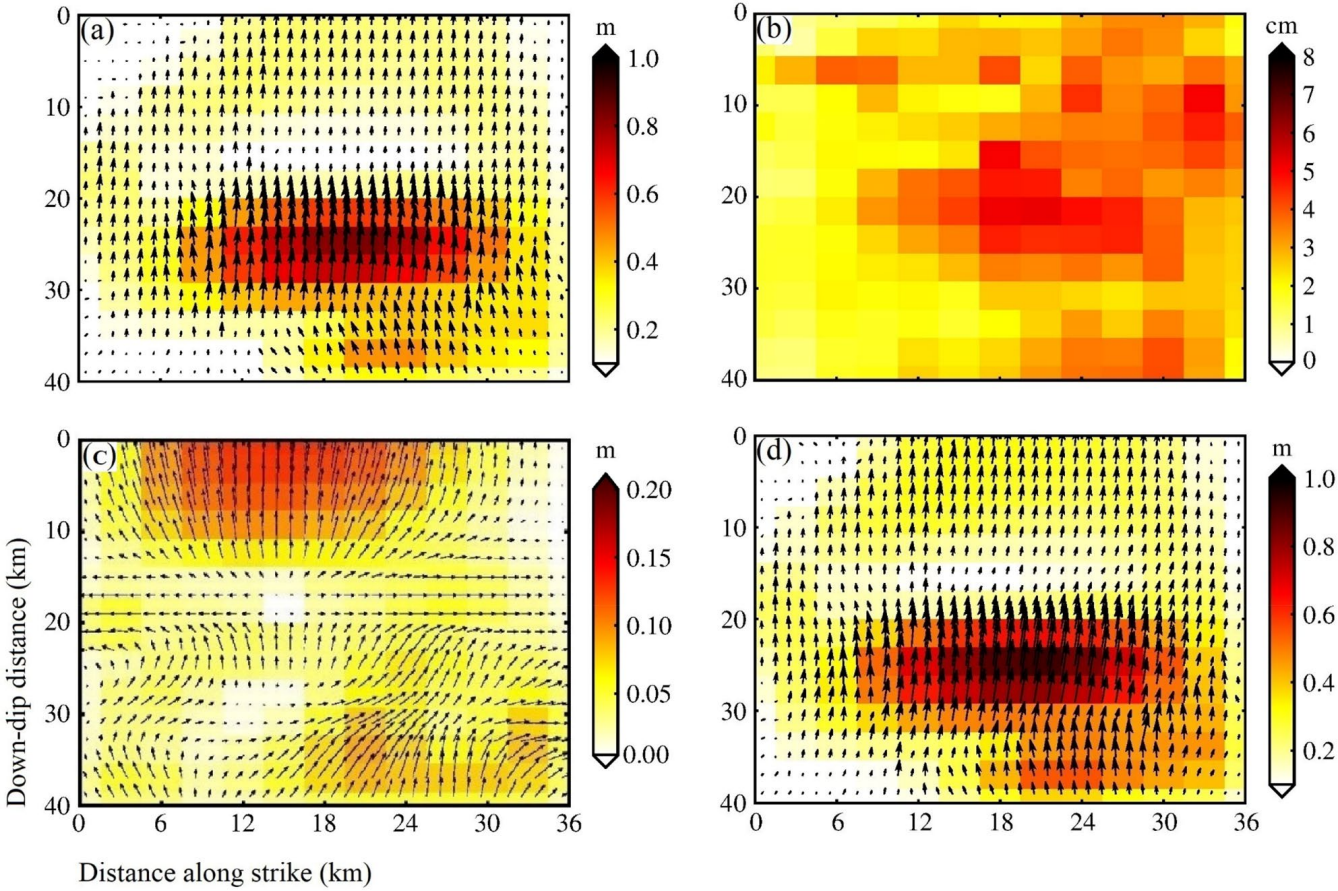
The distribution of the co-seismic and the post-seismic slips along the fault. (**a**) The co-seismic slip distribution; (**b**) The standard deviation of the slip distribution; (**c**) The post-seismic after-slip distribution at 447 days; (**d**) The slip distribution of the sum of (**a**) and (**c**). The horizontal axis and vertical axis are fault length along with the strike and fault depth along with the dip, respectively. The length and the direction of the arrows indicate the magnitude and the direction of slip, respectively.

The fault was enlarged to 36 km strike direction, to 40 km down-dip direction. The fault plane was subdivided, along-strike and down-dip separately, to 1 $\times$ 2 km rectangular pieces. Then, the L-curve method was adopted to solve the smoothing factor. It was eventually determined to be 0.04 (Fig. S3). The fine slip distribution, for the co-seismic rupture fault of the Pishan Mw6.5 earthquake, was simulated by the SDM method, as shown in Fig. 7a. The SDM program calculated that the correlation coefficient was 99.3%, to balance the prediction and observation. To score the reliability, Gaussian noise is applied to the observation data to generate 100 perturbed datasets for the fault slip distribution^11^. We calculated standard deviation of the slip distribution with 100 perturbed datasets, as shown in Fig. 7b. The average errors of slip are ~ 4 cm, and the maximum error appears in the middle of the model up to 6 cm. The slip distribution errors have no essential influence on the overall slip distribution, it was indicated that our consequences are credible in the paper.

At a dip depth of 24–27 km, 22–23 km width, the remarkable slip was embodied a distinct feature, to the top magnitude of 0.9 m. At 0–7 km of the upper layer closed to the value of ~ 0.2 m, there has a nearly uniform slip (Fig. 7a). However, an absence for the slip between 12.5 and 17.5 km (Fig. 7a) may be due to a slip rate deficit. Although no surface rupture was found by the Institute of Geology, claimed by China Earthquake Administration (CEA), Wu et al. \* MERGEFORMAT^15^ found several tensile-type ground fissures at the top of the seismic anticline, and all of the strikes were the same as the NW direction and the anticline. It is conceived that the amount of dislocation near the deep source is bound up with ground fissures and is gradually transformed into the fold of the stratum during propagation and expansion along the fault from stress in the transition. The geodetic moment was approximate 6.36 N$\times 10^{18}$ m, calculated from the coseismic slip distribution, corresponding to M_W_ 6.5. From M_W_ 6.2 (USGS) to M_W_ 6.5 (CENC) for seismological computations were compared, matched group estimated in Table S2. The geodetic moment of the 447 days after the mainshock, calculated from the afterslip distribution was approximately 2.65 $\times$ 10^17^ N⋅m, which accounts for approximately 4.1% of the co-seismic geodetic moment.

The post-seismic afterslip on September 22, 2016 (447 days after the mainshock) and the coseismic slip of the fault were superimposed, as shown in Fig. 7d. The peak magnitude of afterslip at 447 days after the earthquake is only 0.18 m (Fig. 7c), while the peak magnitude of the co-seismic slip reaches 0.9 m (Fig. 7a). Most of the afterslip zone overlaps with the co-seismic slip zone, but the slip momentum further strengthened based on the co-seismic slip. Our results are consistent with a previous study, which reported that afterslip does not change the characteristics of the fault co-seismic slip distribution^26^. Compared with the characteristics of co-seismic slip, the post-seismic afterslip extended to the shallow part of the north-central section of the fault, and the slip momentum was increased over time at the dip depth SSE of 30–38 km.

## Summary

We presented a spatial–temporal distribution of post-seismic deformations from the M_W_ 6.5 Pishan earthquake on 3 July 2015, which was derived from 9 Sentinel-1A TOPS SAR images from 18 July 2015 to 22 September 2016 using the SBAS-InSAR technique. The evolution process of post-seismic afterslip distribution was inverted by the SDM method. We comprehensively analyzed the relationship between post-seismic slip and co-seismic slip. The main conclusions are as follows:

1. The post-seismic deformation of the Pishan Ms6.5 earthquake was manifested as an uplift in the satellite line-of-sight (LOS) direction in the western portion of the epicenter, which had a significant positive value of up to 25 mm in the satellite LOS direction and a negative value of up to 20 mm in the satellite LOS direction. As of August 3, 2015, 98 aftershocks of Mw $\ge$ 3.0 occurred; subsequently, seismic events decreased sharply. The shape of LOS deformation shows the logarithmic function attenuation law in the time evolution.
2. At 447 days after the mainshock (September 22, 2016), the afterslip fields form one slip center. It was located 5–25 km along the fault strike, 0–10 km along with the dip, with a peak magnitude of 0.18 m. The geodetic moment that was based on the afterslip distribution 447 days after the mainshock was approximate 2.65 N$\times 10^{17}$ m, which was equivalent to the moment magnitude Mw 5.5, accounting for approximately 4.1% of the co-seismic geodetic moment.
3. The post-seismic mechanism was a dominant up-dip of the seismogenic portion of a fault zone. The temporal evolution of the deformation feature in the satellite LOS direction and aftershocks supported the scenario that afterslip on the fault plane was the most likely mechanism. The temporal evolution characteristics of afterslip and aftershocks supported the standpoint that aftershocks are dominated primarily by afterslip.

Our study helps understand the characteristics of regional fault activity. It will lay a foundation for us that understanding the spatial distribution of fault locking, slip rate deficit and stress accumulation, to better realize mainshock-aftershock activity^27^. These will be the focus of future studies.

## Discussion

It has been determined that the Pishan Mw 6.5 earthquake was the result of the N-direction extrusion of the Tarim block in the Tibetan Plateau, and the seismogenic fault was concealed in the Early-Middle Pleistocene^10,28^. Meanwhile, the results of InSAR observations show that uplift occurred in the western part of the epicenter, and subsidence occurred in the southern part of the epicenter. There are obvious differential deformations on both sides of the fault after the earthquake. The strong earthquake activity near the northwestern margin of the Tibetan Plateau (the West Kunlun orogenic belt) and the Tarim Basin has a certain dynamic relationship with the collision between the Indian Plate and the Eurasian plate, so, close attention should be paid to this region.

The co-seismic slip inversion with different InSAR data^11,12,14^ depicted that the focal depth was mainly distributed in 7–15 km. Nonetheless, the post-seismic afterslip extended to 0–10 km in the shallow part of the northern section, and the slip trend was increased overtime at the deep SSE of the fault (Fig. 7b). The slip rate deficit in the lock (or partial lock), which was converted into stress and gradually accumulated until an earthquake (or fault creep)^25^. There was a slight slip between 15 and 17.5 km dip depths of the fault (Fig. 7d), which was related to the slip deficit caused by the tectonic deformation of reverse fault-fold. Local tensile stress was generated at the transition part of fold strata to change the momentum of slip^15^. The real strong seismogenic area was located on the Tekilik fault at the foot of the mountain in the western Kunlun Mountain, where the strain energy was in a locked state^10,15^. The magnitude of the deep afterslip was increased gradually over time, implying that a creeping process from steady-state “secondary” creeping to accelerating “tertiary” creep in the deep of fault. The region may still trigger earthquakes, and the danger of future earthquakes deserves further attention and research.

## Methods

### Data

Thirteen descending Sentinel-1A Terrain Observation with Progressive Scans (TOPS) SAR images (C-band) acquired from D136, from 18 July 2015 to 22 September 2016 covering the earthquake zone, were collected to estimate the LOS deformation time series. The Sentinel-1A Interferometric Wide (IW) imaging mode uses the TOPS method to obtain three sub-bands with a total width of approximately 250 km and resolutions of 5 m × 20 m along with the range and azimuth directions^29,30^. Compared to traditional scanning or spotlight imaging modes, TOPS mode can significantly improve ScanSAR and achieve better image quality^31,32^. Precision ephemeris orbits (https://qc.sentinel1.eo.esa.int) were used for orbital corrections. Elimination of topographic phase effects using 90 m resolution (https://srtm.csi.cgiar.org) Shuttle Radar Topography Mission (SRTM) digital elevation model (DEM) provided by NASA.

### Generation of multiple differential interferograms

We adopted the Small Baseline Subset InSAR (SBAS-InSAR) technique to process 9 Sentinel-1A TOPS SAR images over the research area. SBAS-InSAR is a method that effectively mitigates decorrelation phenomena by analyzing distributed scatters (DS) with high coherence based on an appropriate combination of interferograms^33,^^\* MERGEFORMAT^^34^. It combines pairs of images with multi-master image methods, obtains the time series of surface deformation of each set by using the least square estimation method, and obtains the minimum norm least square solution of the surface deformation rate between image sequences by using singular value decomposition.

Assuming that a set of $N$ + 1 SAR images is obtained by time series $\left( {t_{0} , \ldots ,t_{N} } \right)$ covering the study area, and co-registered with other SAR images using an arbitrary image as the main image. Finally, a total of $M$ differential interferograms are obtained. If $N$ is an odd number, $M$ can be expressed as^35^:

4 $$ \frac{N + 1}{2} \le M \le N\left( {\frac{N + 1}{2}} \right) $$

**Figure 8:**
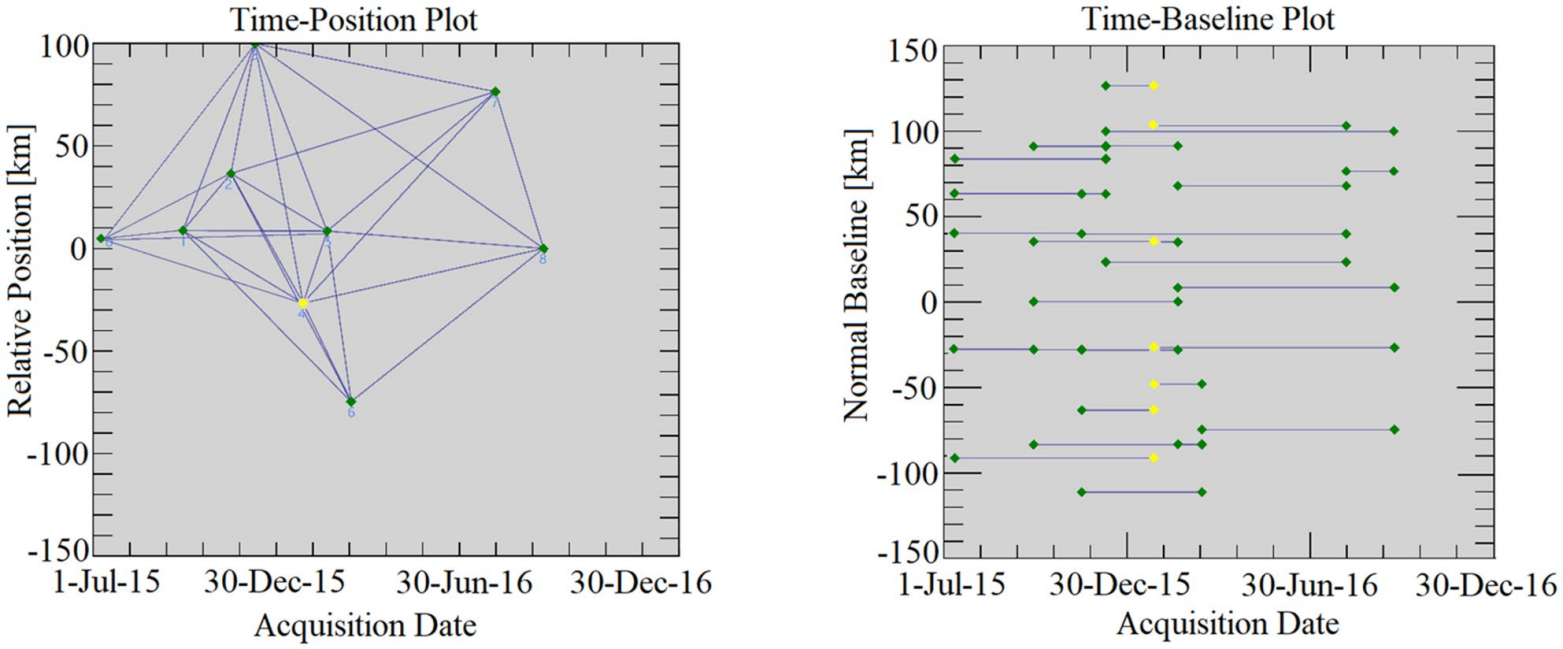
Time-position and time-baseline of Sentinel-1A image interferograms. The yellow diamond denotes the super master image. Green diamonds denote slave images. Blue lines represent interferograms (the Mw6.5 event occurred on 3 July 2015).

We computed a set of multi-look (also referred to, as low-pass (LP) filtered) differential interferograms characterized by constraints on the maximum temporal and perpendicular baseline^36^. We obtained interferograms involving data pairs with Doppler centroid differences within the SAR image Doppler bandwidth. Twenty-eight groups of small baseline interferometric pairs were selected, and the parameters of the interferometric pairs are shown in Table S4. The time baseline distance of all interferograms was less than 300 days. All interferograms are less than 110 m in terms of normal baseline (Fig. 8).

### Atmospheric error correction

Atmospheric components have a strong correlation at short distances^37^. To estimate the atmospheric disturbances, we select high phase coherence (HPC) pixels on the image grid to estimate the atmospheric phase screen (APS), which can be interpolated on the grid. Both operations (filtering and resampling) can be performed at the same time using kriging interpolation^38^.

Estimate the mean value of the estimated atmospheric components in the $H$ HPC pixels of differential interferograms as^38^:

5 $$ APS_{master}^{T} = \frac{1}{K}\left\{ {\underline {1}^{T} \widehat{{ \cdot E_{atmo}^{^{\prime}} }} + \left( {\widehat{{\underline {a} }}\underline {1}^{T} + \underline{{\hat{p}_{\xi } \xi^{T} }} + \underline{{\hat{p}_{\eta } \eta^{T} }} } \right)} \right\} $$

where $APS_{master}^{T}$ is an estimation of the atmospheric phase contribution relative to the master image. $K$ the differential interferograms. $\widehat{{E_{atmo}^{^{\prime}} }}$ contains the residues that include atmospheric effects. $\widehat{{\underline {a} }}\left[ {K \times 1} \right]$ are constant phase values, $\underline{{\hat{p}_{\xi } }}$[$K \times 1$] and $\underline{{\hat{p}_{\eta } }}$[$K \times 1$] contain the slope values of the linear phase components, along the azimuth $\underline{\xi }$[$H \times 1$] and slant range $\underline{\eta }$[$H \times 1$] direction, due to the atmospheric phase contribution.

The phase of each slave image can be modified by these estimated quantities. The new set of phases of the modified slave images will be represented as^38^:

6 $$ \underline{\zeta } = \underline{\Psi } - APS = \underline {1} \underline{{\Psi_{m}^{T} }} + E^{\prime \prime } $$

where $\underline {\Psi } \left[ {K \times H} \right]$ contains the phases of the H HPCs as seen by the $K$ slave images, $\underline{{\psi_{m} }}$[$H \times 1$] contains the phases of the H HPCs as seen by the master image, compensated for $APS_{master}^{T}$, and $E^{\prime \prime } [K \times H]$ is the residue matrix.

### Orbit error correction

Although the orbital parameter correction has been performed on the Sentinel-1A data using the precision ephemeris orbit data before the data interference processing, there are still residual orbit errors. In this paper, the polynomial optimization method is used to remove the residual orbit errors based on the atmospheric delay error correction^39^. Estimate the orbital error phase of a single interference image pair as:

7 $$ R_{ij} \left( {x,y} \right) = d_{ij} \cdot xy + a_{ij} \cdot x + b_{ij} \cdot xy + c_{ij} { } $$

where ${ }ij$ represents the SAR image number that constitutes the interference image pair, $\left( {x,y} \right)$ represents the pixel position of the interferometric image, and $a_{ij}$, $b_{ij}$, $c_{ij}$, $d_{ij}$ represent the orbit error correction coefficients of the interference image pair $ij$. Finally, the orbit error correction coefficient is corrected by the least-squares method to obtain the optimal solution. Fig. S4 shows an example of atmospheric and orbital error correction for interferometry.

### Time series retrieval

The deformation rate was estimated using the least-squares method^40^. Assuming that the terrain phase has been removed by DEM, the interference phase on the i-th point target can be expressed as^41,42^:

8 $$ \varphi_{i}^{k} = wrap\left( {\varphi_{i,defo}^{k} + \varphi_{i,atmo}^{k} + \varphi_{i,orb}^{k} + \varphi_{i,topo - res}^{k} + \varphi_{i,noise}^{k} } \right) $$

where $wrap( \cdot )$ represents the phase wrap operator; $\varphi_{i}^{k}$ represents the interferometric phase observation on the point target; $\varphi_{i,atmo}^{k}$ indicates atmospheric unevenness interference phase; $\varphi_{i,orb}^{k}$ is the interference phase caused by the baseline error; $\varphi_{i,topo - res}^{k}$ is the interference phase caused by the DEM residual; and $\varphi_{i,noise}^{k}$ indicates a loss of coherent noise.

The phase of the interference $\varphi_{i,defo}^{k}$ can be expressed as a polynomial model, as shown in the following equation^42,43^:

9 $$ \varphi_{i,defo}^{k} = \frac{4\pi }{\lambda }\mathop \sum \limits_{p = 1}^{N} \left( {t_{i}^{k} } \right)u_{i,p} + \varphi_{i,res - def}^{k} $$

where $u_{i,p}$ correspond to linear rate acceleration and cubic velocity terms in the N-order polynomial deformation model, respectively; $t_{i}^{k}$ is the image time difference of the kth interferogram. λ is the radar wavelength. $\varphi_{i,res - def}^{k}$ is the residual phase of the interferogram $i$ after the N-order polynomial simulation; it will be a small quantity. Theoretically, ground deformation is seen as a continuous function over time and can be uniformly approximated by polynomials^41^.

There is a linear relationship between $\varphi_{i,topo - res}^{k}$ and DEM residual $\Delta h_{i}$, which can be expressed as^44^:

10 $$ \varphi_{i,topo - res}^{k} = \frac{4\pi }{\lambda } \times \frac{{B_{ \bot ,i}^{k} \Delta h_{i} }}{{rsin\beta_{i} }} $$

where λ is the radar wavelength; $B_{ \bot ,i}^{k}$ is the vertical baseline distance of the kth interferogram, and r is the slant distance between the ground point and sensor; $\beta_{i}$ is the angle of incidence.

The interference phase model can be further expressed as:

11 $$ \varphi_{i}^{k} = \frac{4\pi }{\lambda } \times \frac{{B_{ \bot ,i}^{k} \Delta h_{i} }}{{rsin\beta_{i} }} + \frac{4\pi }{\lambda }\mathop \sum \limits_{p = 1}^{N} \left( {t_{i}^{k} } \right)u_{i,p} + \varphi_{i,res - def}^{k} + \varphi_{i,atmo}^{k} + \varphi_{i,orb}^{k} + \varphi_{i,noise}^{k} $$

The $\varphi_{i,noise}^{k}$ can be eliminated by the phase difference operation between two adjacent point targets^42^. The high-order polynomial simulates the deformation process;$\varphi_{i,res - def}^{k}$ will be small, which will ensure that Eq. (9) is established and the correct model solution is obtained. In general, a third-order polynomial is a robust choice^42^. We obtain the deformation time series by solving the above interferometric phase model.

### Inversion of post-seismic afterslip distribution

To reduce the amount of inversion calculation and improve the signal-to-noise ratio of the data, we first performed a quad-tree downsampling processing on the post-seismic deformation fields for the 8 dates, and the number of deformation points is reduced from 21,927 to 967. Each date of the post-seismic deformation fields had the same number of observation points after downsampling, which is beneficial for comparison in the afterslip inversion. In the case of quadtree downsampling, the sampling in the near-field region was relatively dense, and the sampling in the far-field region was relatively sparse; therefore, the high-resolution information of the deformation field near the epicenter was obtained to the greatest extent.

The fault model is an important parameter for post-seismic afterslip inversion^26^. In general, the fault parameters of the afterslip inversion can refer to the fault parameters of the co-seismic deformation nonlinear inversion^43^. The parameters of the fault model were set by referring to the existing co-seismic fault model parameters (Table S2) and combining the geometric image features of the co-seismic deformation fields and the post-seismic deformation fields.

Assume that the fault plane passes through the epicenter and its upper boundary reaches the surface. The length of the fault (in the direction of the strike) is extended to 36 km, and the lower boundary is extended downward (in the direction of the dip) to 40 km along the fault plane. According to the focal mechanism solutions given by USGS, GCMT, and CENC, the range of the fault strike change is set between 95° and 125°, the range of the dip change is set to 18° to 35°, and a search calculation is performed every 1°. After many inversion experiments, the combination of strike and dip angles with minimum root mean square error of fitting residual and the highest modulus correlation coefficient was selected as the optimal value^44^. Finally, the strike and dip angles of the co-seismic faults were selected as 115° and 25°, respectively.

The SDM program developed by Professor Wang Rongjiang is used to constrain the post-earthquake afterslip. The SDM program uses the steepest descent method for inversion calculation, which is an iterative algorithm for constrained least-squares optimization^45^. When the fault geometry parameters are determined, the slip parameters on the fault plane and the InSAR observed deformation values can be transformed into general linear problems. The expression is:

12 $$ d = Gm + \varepsilon $$

where $d$ is the LOS InSAR observation; $G$ is the Green's function calculated using the dislocation theory according to the elastic semi-infinite space model; $m$ is the momentum of slip on the fault plane; $\varepsilon$ represents the error of the observed data.

To obtain a more detailed fault slip distribution, the fault plane was divided into discrete rectangular elements of 1 km $\times$ 2 along-strike and down-dip, and the medium Poisson's ratio parameter was set to 0.25. We adopted the weight ratio using the residual root mean square (RMS) of each dataset to weigh the contributions of two datasets, and the final weight ratio was 1:0.75. To avoid oscillation or excessive smoothing of the inversion results, Laplace smoothing constraints were added:

13 $$ F\left( m \right) = \left| {\left| {Gm - y} \right|} \right|^{2} + {\text{a}}^{2} \left| {\left| {H\tau } \right|} \right|^{2} $$

where $m$ represents the amount of slip on each sub-fault plane of the fault, $G$ is the Green's function, $y$ represents the LOS InSAR observation, ${\text{a}}$ represents the smoothing factor, weighing the fit of the observed data and the roughness of the inversion result, $H$ denotes the finite difference approximation expression of the Laplacian operator multiplied by a weighting factor proportional to the relevant slip, and $\tau$ indicates the shear stress drop linearly related to the slip distribution on the fault plane.

The smoothing constraint was applied to the slip distribution to overcome the instability of the inversion results^20^. The smoothing factor is generally obtained by plotting the compromise curve between the roughness and the misfit of the test data. The determined optimum smoothing factor was 0.04 using an L-curve plot (Fig. S3). Meanwhile, the layered earth structure model derived from Crust1.0 A^46^ model was used for fault slip inversion, and the model parameters are shown in Table S3. As a consequence, the fault model was used to invert the afterslip distribution of the remaining 8 dates of the post-seismic deformation fields.

## Supplementary information

Supplementary Information.
