# Supplementary material for: Post-seismic deformation mechanism of the July 2015 MW 6.5 Pishan earthquake revealed by Sentinel-1A InSAR observation
Source: Sci Rep. 2020 Oct 28;10:18536. doi: 10.1038/s41598-020-75278-0 (PMC7595191; doi:10.1038/s41598-020-75278-0)
Supplement: Supplementary file 1 — Supplementary Information. [file 41598_2020_75278_MOESM1_ESM.pdf]

# Supplementary Information

## Post-seismic Deformation Mechanism of the July 2015 $M_W$ 6.5 Pishan Earthquake Revealed by Sentinel-1A InSAR Observation

Sijia Wang [1*], Yongzhi Zhang[1,2*], Yipeng Wang[1], Jiashuang Jiao[1], Zongtong Ji[1], and Ming Han[1]

[1] College of Geology Engineering and Geomatics, Chang'an University, Xi'an 710054, China

[2] State Key Laboratory of Geo-information Engineering, Xi'an 710054, China

# General information

**Table S1**. Details of co-seismic data were used in this study.

| Orbit | Path | Acquisition | Frame | $B_{\perp}$(m) | $\Delta$t(days) | Inc. Angle(°) | Azi. Angle(°) |
|---|---|---|---|---|---|---|---|
| **Ascending** | 056 | 20150630-20150724 | 120 | -53 | 24 | 33.7 | -169.3 |
| **Descending** | 136 | 20150624-20150718 | 465 | 26 | 24 | 41.4 | -9.8 |
| **Descending** | 136 | 20150624-20150718 | 470 | 26 | 24 | 41.4 | -9.8 |

**Table S2.** Source parameters of the 2015 Pishan earthquake.

| Source | Lon. /(°) | Lat. /(°) | Strike /(°) | Dip /(°) | Length km | Width km | Slip m | $M_W$ |
|---|---|---|---|---|---|---|---|---|
| **USGS [a]** | 78.154 | 37.459 | 98 | 34 | - | - | - | 6.2 |
| **GCMT [b]** | 78.14 | 37.58 | 109 | 22 | - | - | - | 6.4 |
| **CENC [c]** | 78.1 | 37.5 | 115 | 23 | - | - | - | 6.5 |
| **Wen et al.** | 78.057 | 37.571 | 114 | 25 | 36 | 40 | 0.34 | 6.5 |
| **Uniform slip model** | 78.03 ±0.0004 | 37.50 ±0.0005 | 114.0 ±1.6 | 24 ±1.2 | 22.6 ±1.5 | 10.2 ±1.4 | 0.56 ±0.07 | 6.4 |
| **This study** | 78.03 | 37.50 | 114.0 | 25 | 36 | 40 | 0.35 | 6.5 |

[a] United States Geological Survey. [b] Global Centroid Moment Tensor Catalogue. [c] China Earthquake Network Center

**Table S3**. The Crust1.0 crustal layered earth structure

| layer | Depth/ km | $V_P$/ km·s$^{-1}$ | $V_S$/ km·s$^{-1}$ | Rho(ρ) / kg·m$^{-3}$ | η/ Pa·s |
|---|---|---|---|---|---|
| **1** | 0.00 | 4.0000 | 2.1300 | 2370 | ∞ |
| **2** | 3.39 | 4.0000 | 2.1300 | 2370 | ∞ |
| **3** | 3.39 | 5.0000 | 2.8800 | 2540 | ∞ |
| **4** | 3.89 | 5.0000 | 2.8800 | 2540 | ∞ |
| **5** | 3.89 | 6.1000 | 3.5500 | 2740 | ∞ |
| **6** | 23.91 | 6.1000 | 3.5500 | 2740 | ∞ |
| **7** | 23.91 | 6.3000 | 3.6500 | 2780 | $1\times 10^{19}$ |
| **8** | 41.65 | 6.3000 | 3.6500 | 2780 | $1\times 10^{19}$ |
| **9** | 41.65 | 7.0000 | 3.9900 | 2950 | $1\times 10^{19}$ |

**Table S4**. Details of post-seismic interferometric pairs were used in this study.

| **No.** | **Acquisition** | **B(km)** | **$\Delta$t(days)** |
|---|---|---|---|
| **1** | 20150928-20150718 | -30.8650 | 72 |
| **2** | 20150928-20151209 | 93.5020 | 72 |
| **3** | 20150928-20160314 | -81.1070 | 168 |
| **4** | 20151115-20150718 | 63.6750 | 120 |
| **5** | 20151115-20150928 | -32.14 | 48 |
| **6** | 20151115-20151209 | 63.5330 | 24 |
| **7** | 20151115-20160126 | -63.2924 | 72 |
| **8** | 20151115-20160219 | -28.2582 | 96 |
| **9** | 20151115-20160314 | -110.1997 | 120 |
| **10** | 20151115-20160805 | 42.3546 | 264 |
| **11** | 20160126-20150718 | 100.3620 | 192 |
| **12** | 20160126-20150928 | 34.0432 | 120 |
| **13** | 20160126-20151209 | 126.2527 | 48 |
| **14** | 20160126-20160219 | 34.5508 | 24 |
| **15** | 20160126-20160314 | -47.4389 | 48 |
| **16** | 20160126-20160805 | 102.8146 | 192 |
| **17** | 20160219-20150718 | 70.2860 | 216 |
| **18** | 20160219-20150928 | -8.2758 | 144 |
| **19** | 20160219-20151209 | 91.7461 | 72 |
| **20** | 20160219-20160314 | -81.9811 | 24 |
| **21** | 20160219-20160805 | 69.4629 | 168 |
| **22** | 20160805-20151209 | 26.2252 | 240 |
| **23** | 20160805-20170508 | -18.5854 | 276 |
| **24** | 20160922-20151209 | 99.8783 | 288 |
| **25** | 20160922-20160126 | -26.6835 | 240 |
| **26** | 20160922-20160219 | 8.4833 | 216 |
| **27** | 20160922-20160314 | -74.6694 | 192 |
| **28** | 20160922-20160805 | 76.4581 | 48 |

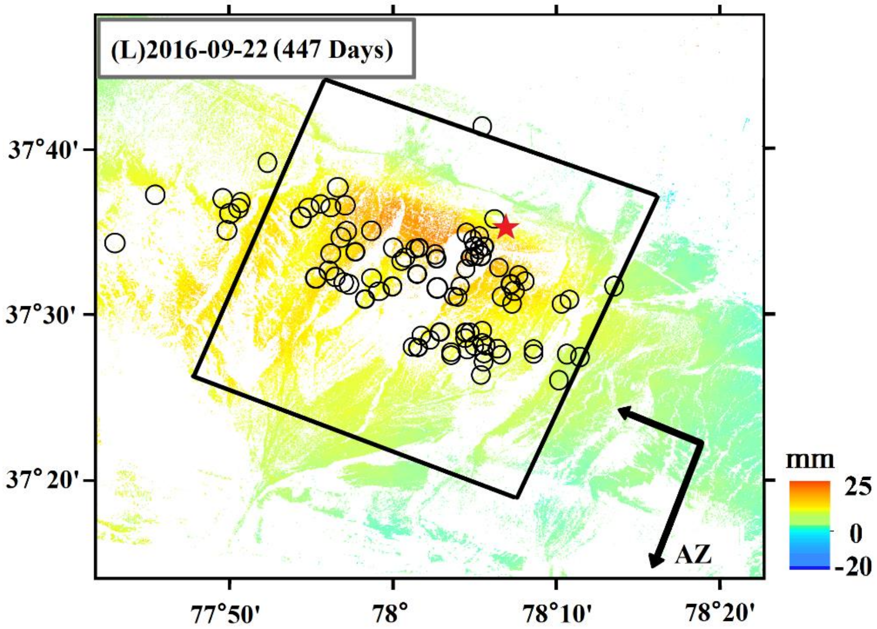


**Figure S1.** The cumulative LOS deformation from 15 November 2015 to 22 September 2016. Positive range change represents motion towards away from the satellite, while negative range change indicates motion away from the satellite. The red star represents the epicenter. The black circles indicate aftershocks (Mw≥3.0) up to 447 days after the mainshock. The black rectangle represents the surface projection of the modeled fault.

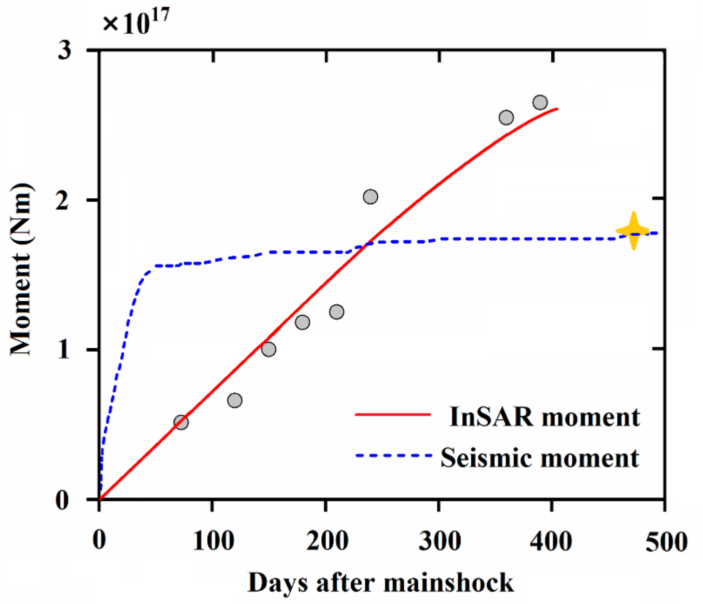


**Figure S2**. Time evolution of cumulative post-seismic moment released by afterslip (grey dots, with best-fitting exponential curve marked by the red line) and aftershocks (blue dashed line). The yellow star indicates the aftershock (Mw≥3.0) on October 5, 2016.

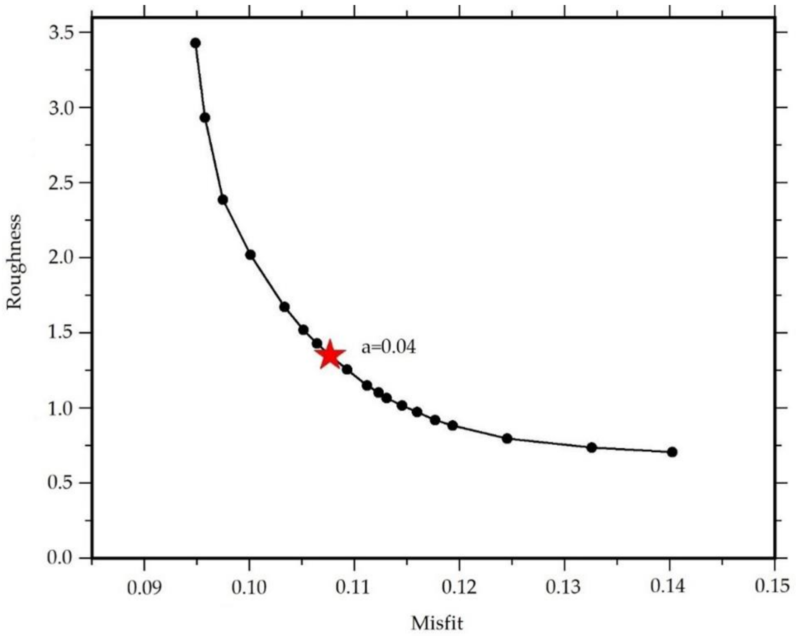


**Figure S3.** The curve representing the tradeoff between the model roughness and misfit. The red star indicates the location of the optimal smoothing parameter.

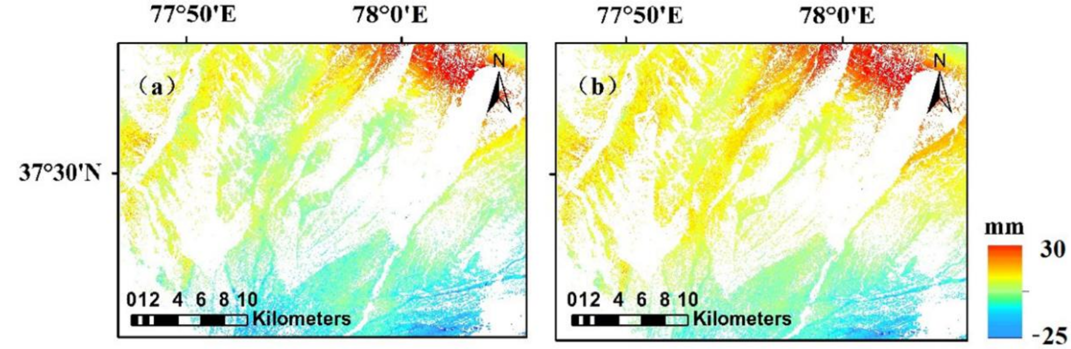


**Figure S4.** An example of atmospheric and orbital error correction for interferometry. (a) The uncorrected interference deformation image; (b) The image after atmosphere correction and orbit error correction.
